# Supplementary material for: Emerging strains of watermelon mosaic virus in Southeastern France: model-based estimation of the dates and places of introduction
Source: Sci Rep. 2021 Mar 29;11:7058. doi: 10.1038/s41598-021-86314-y (PMC8007712; doi:10.1038/s41598-021-86314-y)
Supplement: Supplementary file 1 — Supplementary Information [file 41598_2021_86314_MOESM1_ESM.pdf]

SUPPLEMENTARY INFORMATION

Emerging strains of watermelon mosaic virus in Southeastern France: model-based estimation of the dates and places of introduction

L Roques[1,*], C Desbiez[2], K Berthier[2], S Soubeyrand[1], E Walker[1], E K Klein[1], J Garnier[3], B Moury[2], J Papaïx[1]

[1]INRAE, BioSP, 84914 Avignon, France
[2]INRAE, Pathologie Végétale, F-84140, Montfavet, France
[3]Laboratoire de Mathématiques (LAMA), CNRS and Université de Savoie-Mont Blanc, Chambéry, France
[*] lionel.roques@inrae.fr

Supplementary Fig. S1. Effect of the initial population density

We computed the proportions of each strain with the MLE $\mathbf{\Theta}^*$, assuming other initial densities of the ESs instead of 1/10th of the carrying capacity at the introduction point. **Fig. S1** depicts the proportions of each strain, assuming either that the initial density of the ESs are increased or decreased by a factor 2.

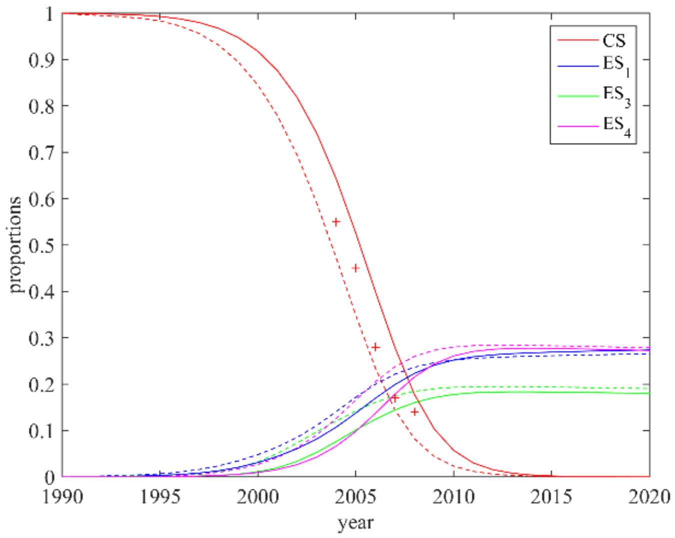

Fig. S1. Estimated average proportions of the classical and emerging strains in the study area with varying values of the initial density of the ESs. The plain lines correspond to initial densities divided by 2 (1/20th of the carrying capacity) and the dotted lines to initial densities multiplied by 2 (1/5th of the carrying capacity).

Supplementary Fig. S2. Finite element method.

Partial differential equations were solved with Comsol Multiphysics time-dependent solver which is based on a finite element method (FEM). The triangular mesh which was used for our computations is depicted below (**Fig. S2**). It is made of 4706 triangular elements.

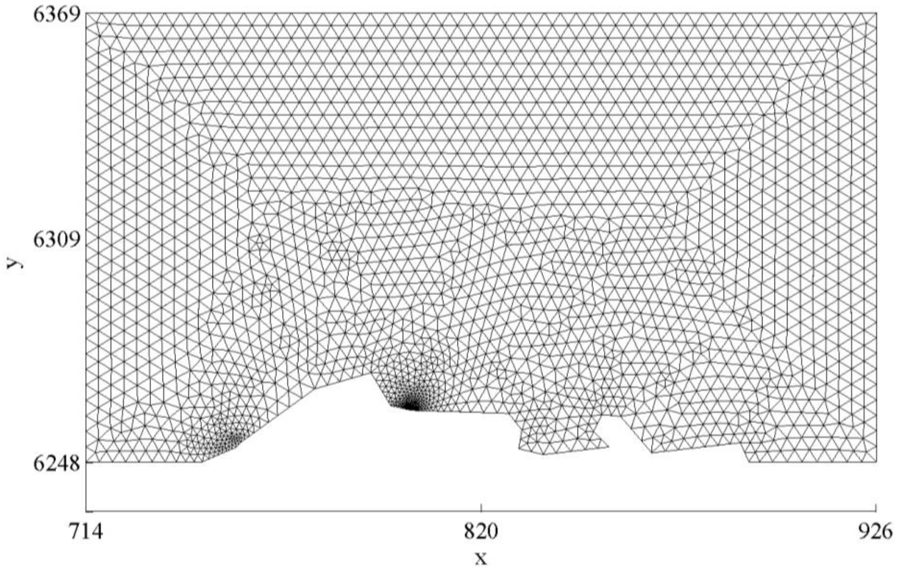

Fig. S2. Triangular mesh of the study site.

Supplementary Fig. S3: Introduction points

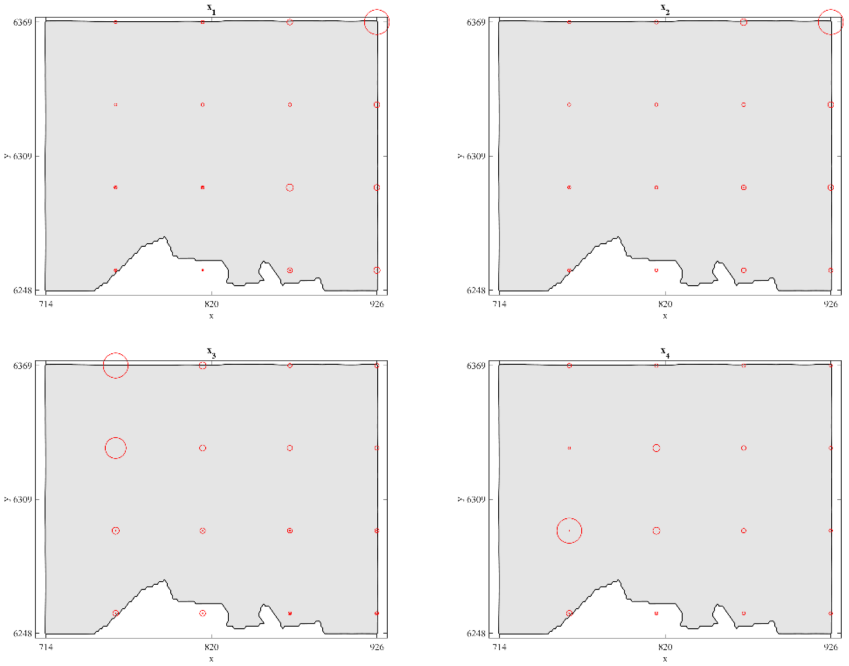

Fig. S3. Likelihood function in terms of the introduction point. The area of the circles are proportional to the highest value reached by the function $f(\mathbf{\Theta}_j)$ when the introduction point of the ES_k (each panel corresponds to a different strain) is located at the center of the circle.

Supplementary Fig. S4: Profile of the likelihood function.

The simulated annealing algorithms led to 6 sequences $(\boldsymbol{\Theta}_j)_{j\geq 1} \in \mathbb{R}^{16}$, for a total of 32000 elements $\boldsymbol{\Theta}_j$ and 32000 evaluations of $\mathcal{L}(\boldsymbol{\Theta}_j)$. Fig. S4 depicts the values of a monotone transform of the likelihood $f(\boldsymbol{\Theta}_j) = \frac{100}{100+\log(\mathcal{L}(\boldsymbol{\Theta}^*)/\mathcal{L}(\boldsymbol{\Theta}_j))}$, projected onto the 1D variables D, r, m_c, m_e, n_1, n_2, n_3, n_4. We observe that close parameter values tend to lead to close values of the likelihood, which strongly suggests that the MLE $\boldsymbol{\Theta}^*$ is close to the actual maximizer of the likelihood function.

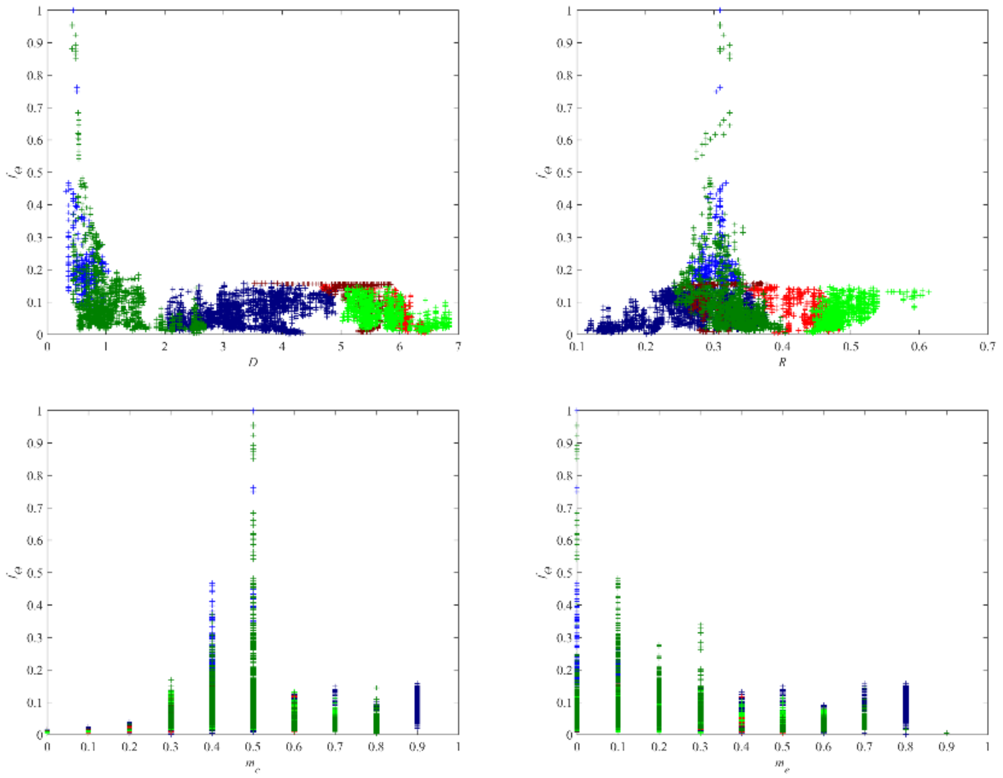

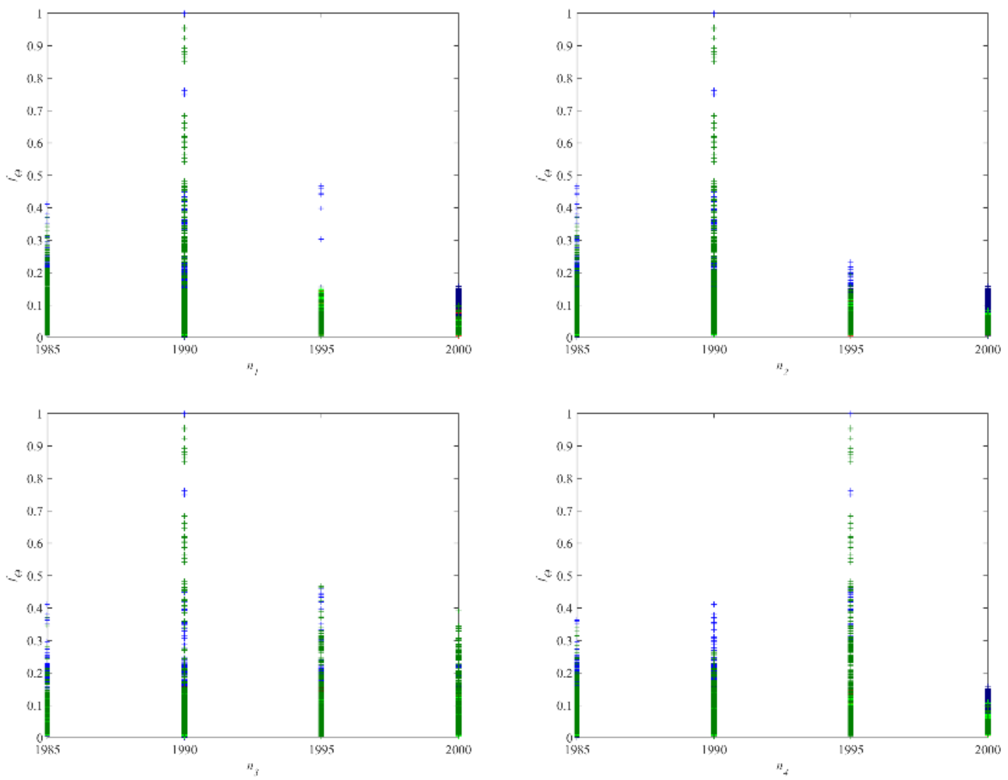

Fig. S4. Profile of the likelihood function. Each panel corresponds to a projection of the 32000 computed values of $f\left(\mathbf{\Theta}_j\right)$, over the 1D variables D, r, m_c, m_e, n_1, n_2, n_3, n_4, respectively. The blue crosses corresponds to parameters $\mathbf{\Theta}_j$ obtained with the slowest cooling rate ($\alpha = 0.9995$, 2 chains); the green crosses to the intermediate cooling rate ($\alpha = 0.999$, 2 chains) and the red crosses correspond to fastest cooling rate ($\alpha = 0.995$, 2 chains). Note that $f(\mathbf{\Theta}^*) = 1$ and $f(\mathbf{\Theta}) = 0.1$ when $\log\left(\mathcal{L}(\mathbf{\Theta})\right) = \log\left(\mathcal{L}(\mathbf{\Theta}^*)\right) - 900$ (here, $\log\left(\mathcal{L}(\mathbf{\Theta}^*)\right) \approx -1229$).

Supplementary Fig. S5: Proportions of the classical and emerging strains in the landscape: 2008.

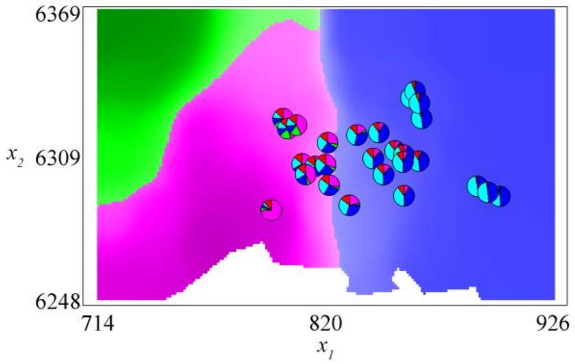

Fig. S5. Proportions of the classical and emerging strains in the landscape: data and simulations. The colors of the shaded regions indicate which strain is the most prevalent. The red regions correspond to the classical strain; light blue and blue: ES_1, ES_2 (these two strains have the same density, only ES_2 is represented); green: ES_3; pink: ES_4. The pie charts describe the relative proportions of the strains found in the data (same color legend). The simulation results presented here correspond to the middle of the intra-annual stage (2nd week of June), and were obtained with the MLE $\mathbf{\Theta}^*$.

Supplementary Fig. S6. Full timeline of the dynamics of the different strain proportions in the landscape, obtained with the maximum likelihood estimate (MLE) Θ^*.

The pictures below represent the dynamics of the classical and emerging strains, from the first estimated introduction date of an emerging strain (1990) to 2019. The colors of the shaded regions indicate which strain is the most prevalent. The red regions correspond to the classical strain; blue: ES_1, ES_2 (these two strains have the same density, only ES_1 is represented); green: ES_3; pink: ES_4. The results presented here correspond to the middle of the intra-annual stage (2nd week of June).

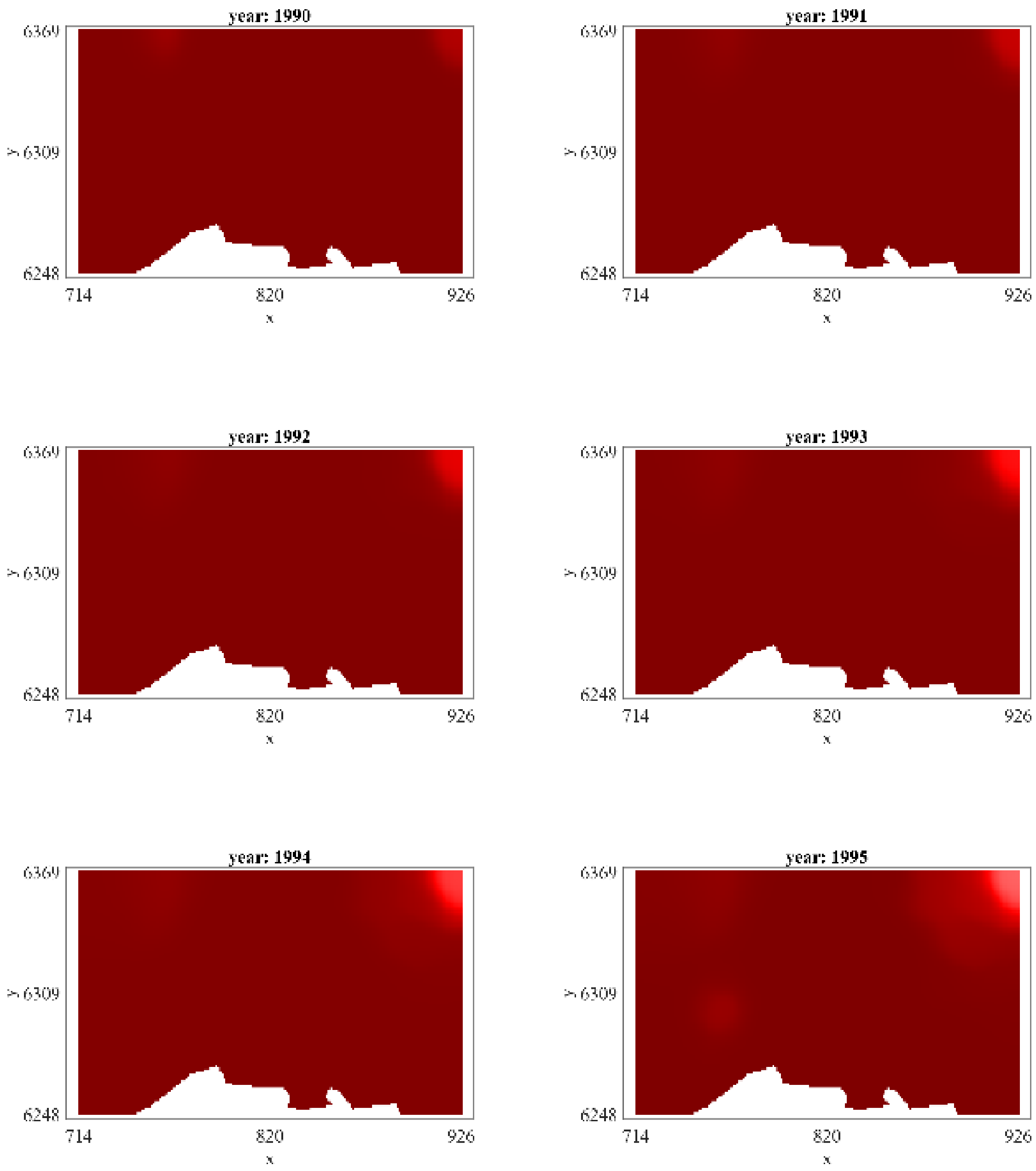

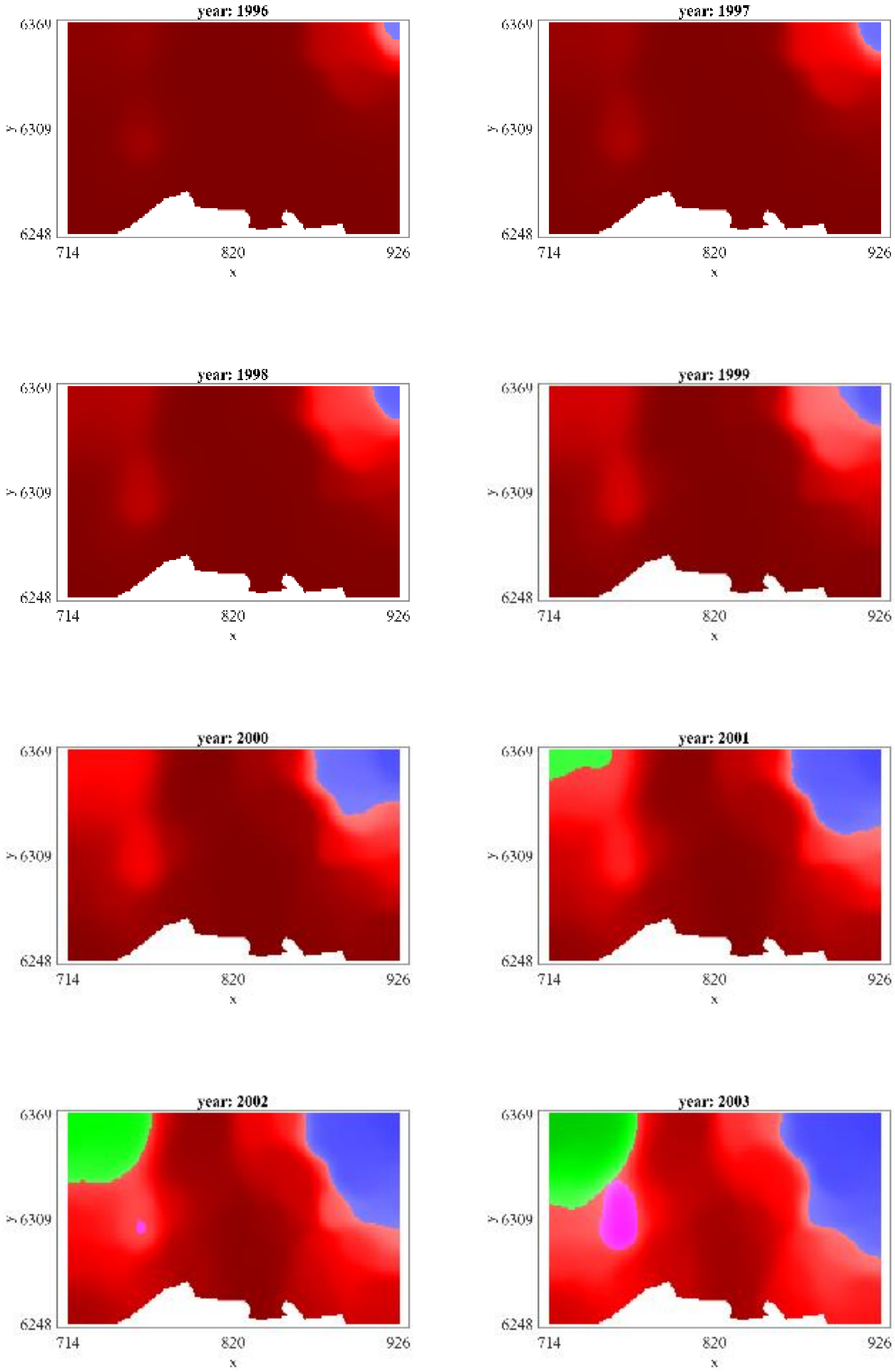

year: 1996
year: 1997
year: 1998
year: 1999
year: 2000
year: 2001
year: 2002
year: 2003
6369
y 6309
6248
714
820
926
x

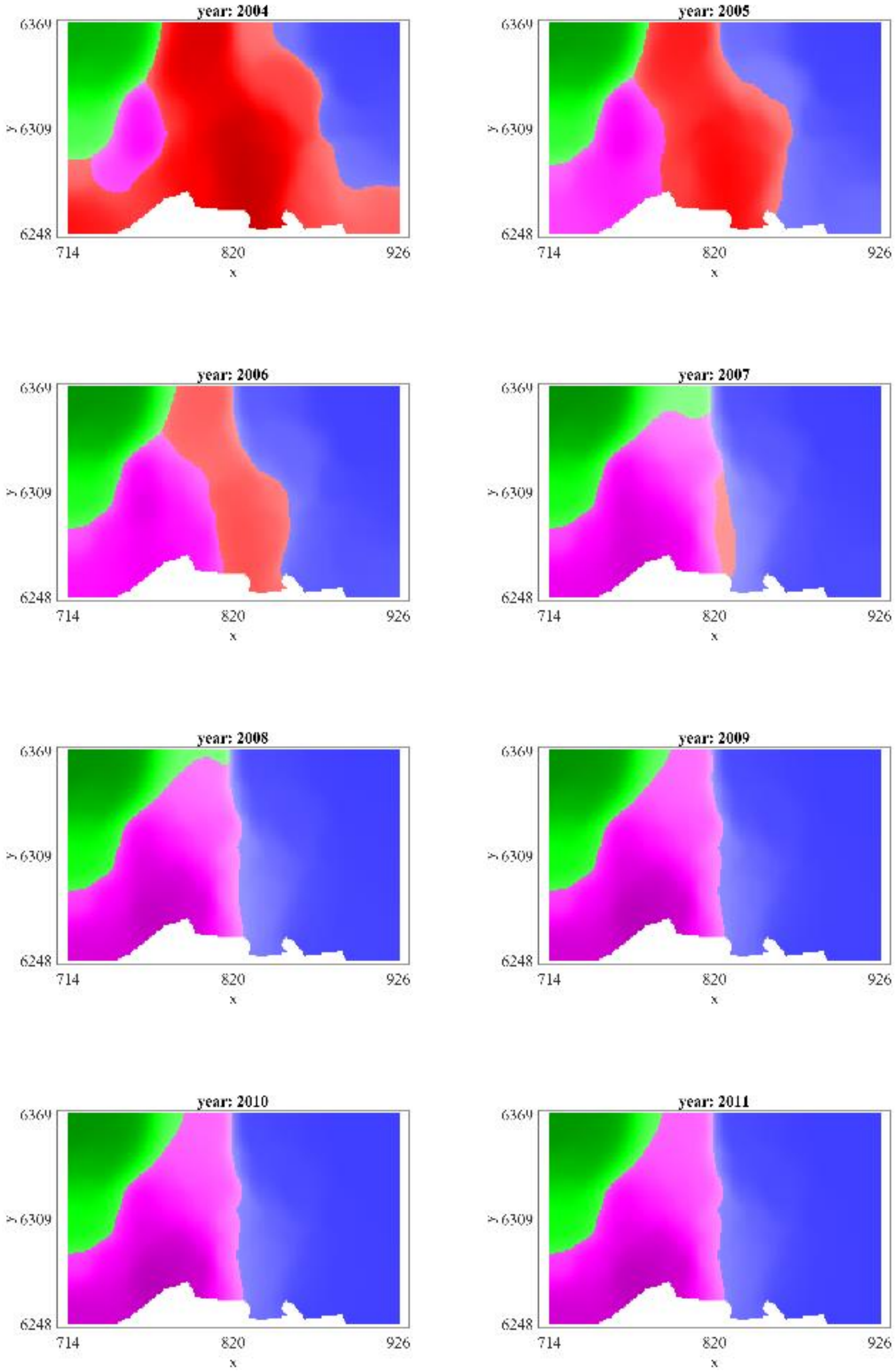
year: 2004
6369
y 6309
6248
714
820
926
x
year: 2005
6369
y 6309
6248
714
820
926
x
year: 2006
6369
y 6309
6248
714
820
926
x
year: 2007
6369
y 6309
6248
714
820
926
x
year: 2008
6369
y 6309
6248
714
820
926
x
year: 2009
6369
y 6309
6248
714
820
926
x
year: 2010
6369
y 6309
6248
714
820
926
x
year: 2011
6369
y 6309
6248
714
820
926
x

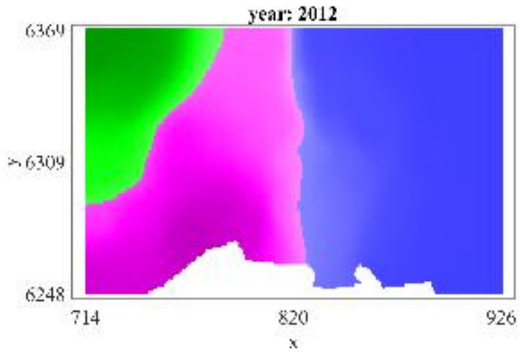
year: 2012
6369
y 6309
6248
714
820
926
x

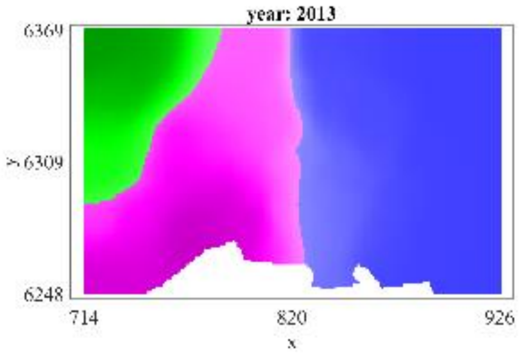
year: 2013
6369
y 6309
6248
714
820
926
x

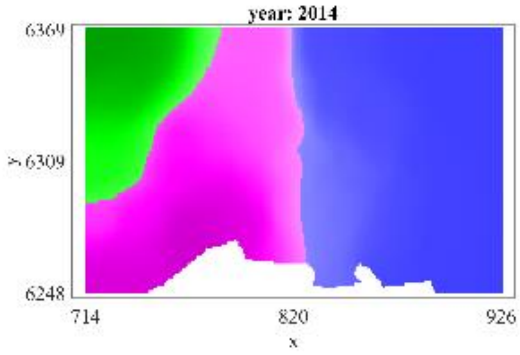
year: 2014
6369
y 6309
6248
714
820
926
x

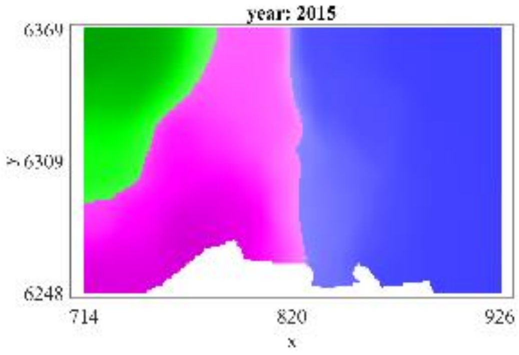
year: 2015
6369
y 6309
6248
714
820
926
x

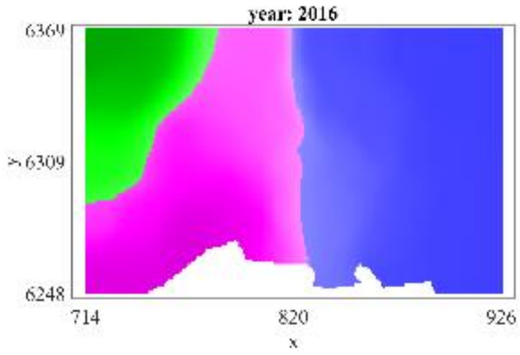
year: 2016
6369
y 6309
6248
714
820
926
x

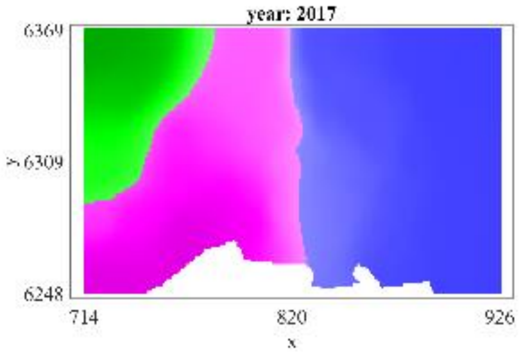
year: 2017
6369
y 6309
6248
714
820
926
x

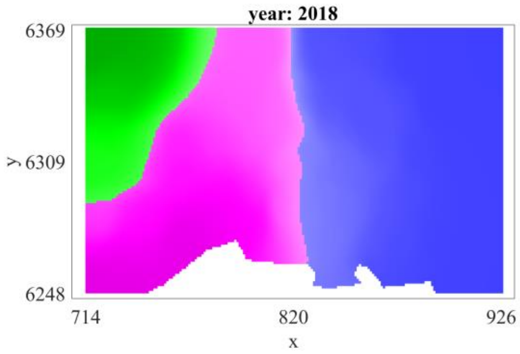
year: 2018
6369
y 6309
6248
714
820
926
x

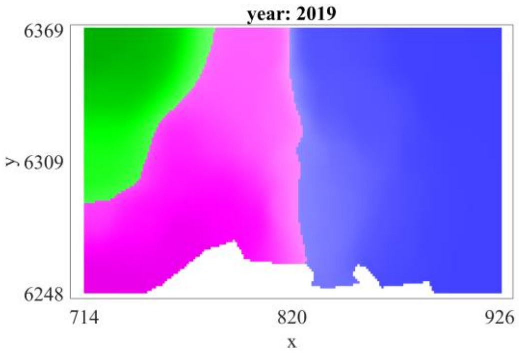
year: 2019
6369
y 6309
6248
714
820
926
x

Supplementary Note S7. A priori bounds on the parameter values, and relationship between diffusion coefficient and dispersal distance

Assuming a random walk movement with discrete space step λ and time step τ, the corresponding diffusion coefficient is $D = \frac{\lambda^2}{4\tau}$. With a time step τ equal to 1 day, we get $\lambda = 2\sqrt{D}$. The bounds $D \in (10^{-4}, 10)\ \mathrm{km}^2/\mathrm{day}$ thus correspond to space steps $\lambda \in (10^{-2}, \sqrt{10})$km. In other words, each day the average distance travelled by a virus (through its vector) is *a priori* assumed to be comprised between 10 m and 3.16 km.

The solution of a pure diffusion equation (i.e., without reproduction), $\partial_t u(t, \boldsymbol{x}) = D\Delta u$ in dimension 2, starting from a localized initial condition at $\boldsymbol{x} = 0$ is $u(t, \boldsymbol{x}) = \frac{1}{4\,\pi D\,t} \exp\left(-\frac{\|\boldsymbol{x}\|^2}{4\,D\,t}\right)$. The mean dispersal distance after 1 day is:

$$\iint \frac{\|\boldsymbol{x}\|}{4\,\pi D\,t} \exp\left(-\frac{\|\boldsymbol{x}\|^2}{4\,D\,t}\right) d\boldsymbol{x} = \sqrt{\pi D}.$$

Having a growth rate r day^{-1} means an increase by a factor e^r each day, in the absence of competition. The bounds $r = 0.1$ and $r = 1$ thus correspond a daily increase by a factor comprised between 1.1 and 2.7.

Supplementary Note S8. Computation of likelihood-ratio based confidence intervals.

To compute confidence intervals for θ_i, where $\boldsymbol{\Theta} = (\theta_1, \dots, \theta_{16})$, we first define the profile likelihood function, $h_i(\rho) = \max_{\boldsymbol{\Theta}\ s.t.\theta_i=\rho} \log(\mathcal{L}(\boldsymbol{\Theta}))$. The $(1-\alpha)$ confidence intervals for θ_i can be constructed by finding the set of parameter values ρ such that $2\left(\log(\mathcal{L}(\boldsymbol{\Theta}^*)) - h_i(\rho)\right) \leq \chi^2_{1-\alpha,1}$, where $\chi^2_{1-\alpha,1}$ is the $(1-\alpha)$ percentile of the χ^2 distribution with 1 degree of freedom [1] (note that $\chi^2_{0.95,1} \simeq 3.84$). For each value of ρ, $h_i(\rho)$ was computed based on the values of $\boldsymbol{\Theta}_j$ which have been explored by the simulated annealing algorithm (using the 6 sequences together, this corresponds to ≈ 32000 values for $\boldsymbol{\Theta}_j$). For the parameters D, r which have not been discretized, to be able to compute $h_i(\rho)$ for values of ρ which have not been explored by the algorithm, we approached $h_i(\rho)$ by $\max_{\boldsymbol{\Theta}\ s.t.\theta_i \in (\rho-\epsilon/2, \rho+\epsilon/2)} \log(\mathcal{L}(\boldsymbol{\Theta}))$, for some $\epsilon \geq 0$ (we took 1/100th of the length of the support of the prior distribution). For the other parameters, $h_i(\rho)$ is computed by interpolation between the discrete grid points.

References

[1] Meeker, W. Q., & Escobar, L. A. (1995). Teaching about approximate confidence regions based on maximum likelihood estimation. *The American Statistician*, *49*(1), 48-53.
